# Supplementary material for: Analysis of Metabolites and Metabolic Pathways of Three Chinese Jujube Cultivar
Source: Metabolites. 2023 May 31;13(6):714. doi: 10.3390/metabo13060714 (PMC10305511; doi:10.3390/metabo13060714)
Supplement: Supplementary file 1 [file metabolites-13-00714-s001.zip › Table S5-S7.pdf]

Table S5 unique differential metabolites in LZ vs MZ

NO.	Metabolite	Class	Chromatographic peak area		VIP	FC	type
			LZ	MZ			
1	Taxifolin(Dihydroquercetin)		1277993	78298	1.06	0.06	down
2	Benzaldehyde		3540267	1054583	1.25	0.3	down
3	Quercetin-3-O-(2''-O-arabinosyl)rutinoside		25115333	8382467	1.24	0.33	down
4	Luteolin-7-O-(6''-sinapoyl)glucoside		87386	30001	1.21	0.34	down
5	Guanine		3039133	1095833	1.25	0.36	down
6	4-O-Glucosyl-4-hydroxybenzoic acid		3785700	1372000	1.25	0.36	down
7	Kaempferol-7-O-glucuronide		1008547	364783	1.23	0.36	down
8	Dambonitol		1820733	668500	1.2	0.37	down
9	L-Prolyl-L-Leucine		1475267	547437	1.24	0.37	down
10	Syringaresinol-4'-O-glucoside		362233	134557	1.24	0.37	down
11	Querspicatin A		3316733	1234700	1.24	0.37	down
12	Kaempferol-3-O-(6''-p-Coumaroyl)glucoside (Tiliroside)		259160	98088	1.26	0.38	down
13	Garbanzol		85988	33919	1.24	0.39	down
14	Aromadendrin (Dihydrokaempferol)		364487	140403	1.24	0.39	down
15	6-O-Caffeoylarbutin		1336033	523343	1.23	0.39	down
16	Cinnamtannin B2		4206300	1648000	1.19	0.39	down
17	24,30-Dihydroxy-12(13)-enolupinol		68164000	27603667	1.26	0.4	down
18	LysoPG 16:0		1129900	448423	1.2	0.4	down

19	Indole-3-Acetaldehyde	7309600	3010000	1.25	0.41	down
20	D-Glucoronic acid	2169667	889773	1.19	0.41	down
21	Betulinic acid	69365333	28248000	1.24	0.41	down
22	Kaempferol-3-O-(2''-O-acetyl)glucuronide	182823	74713	1.25	0.41	down
23	2α-hydroxy-3β-trans-p-hydroxycinnamoyloxy oleanolic acid	3441133	1421033	1.24	0.41	down
24	1-Aminocyclopropane-1-carboxylic acid	108959	45379	1.17	0.42	down
25	N-Acetyl-D-galactosamine	790143	332217	1.19	0.42	down
26	Ursolaldehyde	1110840	462037	1.18	0.42	down
27	Morin	392477	173933	1.22	0.44	down
28	Eriodictyol-7-O-Rutinoside (Eriocitrin)	347943	152723	1.21	0.44	down
29	Sinapoylputrescine	366543	165470	1.24	0.45	down
30	β-Pseudouridine	463973	211873	1.25	0.46	down
31	2R-hydroxy-9Z,12Z,15Z-octadecatrienoic acid	286507	131703	1.17	0.46	down
32	Kaempferide-3-O-glucuronide	86399	40108	1.13	0.46	down
33	Salicin	244853	116054	1.11	0.47	down
34	N-Oleoylethanolamine	424797	198730	1.22	0.47	down
35	Eucommioside	89716	42246	1.2	0.47	down
36	LysoPE 15:1	225193	106250	1.21	0.47	down
37	N-Acetylcadaverine	152280	72521	1.16	0.48	down
38	zizyberenalic acid	482827	230517	1.21	0.48	down
39	Diosmetin-7-O-(6''-malonyl)glucoside	437337	210060	1.21	0.48	down

40	N1,N10-Bis(p-coumaroyl)spermidine		355297	174840	1.17	0.49	down
41	Apigenin-7-O-(6''-malonyl)glucoside		142843	69803	1.23	0.49	down
42	12-Hydroxydodecanoic acid	Lipids	48947	106244	1.12	2.17	up
43	N7-Methylguanosine	Nucleotides and derivatives	532130	1162047	1.17	2.18	up
44	Methylenesuccinic acid	Organic acids	149277	334087	1.1	2.24	up
45	LysoPE 14:0(2n isomer)	Lipids	370353	830420	1.23	2.24	up
46	S-(5'-Adenosyl)-L-methionine	Amino acids and derivatives	12448333	28564333	1.21	2.29	up
47	Kaempferol-7-O-glucoside	Flavonoids	343073	835543	1.24	2.44	up
48	Methyl 3-O-Methyl Gallate	Phenolic acids	35151	86352	1.24	2.46	up
49	Galangin-7-O-glucoside	Flavonoids	212877	532970	1.16	2.5	up
50	10,16-Dihydroxypalmitic acid	Lipids	124400	313760	1.23	2.52	up
51	9,16-Dihydroxypalmitic acid	Lipids	4811333	12364000	1.23	2.57	up
52	Trans dehydrorosinone	Others	294817	762377	1.23	2.59	up
53	LysoPC 14:0	Lipids	4571567	12141667	1.25	2.66	up
54	5'-Deoxy-5'-(methylthio)adenosine	Nucleotides and derivatives	4489167	12223667	1.25	2.72	up

Chromatographic peak area is the relative content of metabolites, chromatographic peak area = average; VIP value represents the variable importance projection value; FC stands for differential multiple.

Table S6 unique differential metabolites in LZ vs STZ

NO.	Metabolite	Chromatographic peak area		VIP	FC	type
		LZ	STZ			
1	8-Azaguanine	5280133	1649533	1.08	0.31	down
2	Sideretin (5,7,8-Trihydroxy-6-methoxycoumarin)	233757	72868	1.11	0.31	down
3	L-Prolyl-L-Phenylalanine	312117	98065	1.24	0.31	down
4	Xanthine	1384300	473773	1.1	0.34	down
5	Coniferin	15963000	5518233	1.25	0.35	down
6	Nystose	111320	39342	1.26	0.35	down
7	Naringenin (5,7,4'-Trihydroxyflavanone)	69371	25293	1.19	0.36	down
8	DL-2-hydroxystearic acid	426843	165130	1.26	0.39	down
9	2R-Hydroxyoctadecanoic Acid	413900	163433	1.26	0.39	down
10	2,5-Dihydroxybenzaldehyde	1753633	707643	1.23	0.4	down
11	Salicylic acid	1727067	705127	1.23	0.41	down
12	Genipin	302770	130983	1.26	0.43	down
13	3-O-trans-p-Coumaroyltormentic acid	469197	199560	1.26	0.43	down
14	4-Hydroxybenzoic acid	2391067	1040540	1.23	0.44	down
15	Hesperetin-7-O-(6''-malonyl)glucoside	183037	80087	1.21	0.44	down
16	6-C-Glucosyl-2-Hydroxynaringenin	18015667	8060967	1.18	0.45	down
17	Aromadendrin-7-O-glucoside	17018000	7722033	1.23	0.45	down
18	D-Saccharic acid	6003300	2779367	1.09	0.46	down
19	LysoPE 18:2	19415667	8938600	1.28	0.46	down
20	Isoceanothic acid	20896667	9644233	1.03	0.46	down
21	Hypoxanthine	1602067	747333	1.14	0.47	down
22	LysoPE 16:1	180960000	84983000	1.28	0.47	down
23	Indole-5-carboxylic acid	409843	197150	1.27	0.48	down

24	Methyl dioxindole-3-acetate	566403	274550	1.24	0.48	down
25	D-Maltotetraose	97931	46628	1.22	0.48	down
26	L-Homocystine	126563	62270	1.23	0.49	down
27	Ferulic acid-4-O-glucoside	1452067	716370	1.28	0.49	down
28	Epicatechin-3'-O-β-D-glucopyranoside	14363667	7050867	1.06	0.49	down
29	LysoPC 18:3	4630100	2277033	1.19	0.49	down
30	LysoPC 18:3(2n isomer)	4630100	2277033	1.19	0.49	down
31	Luteolin-7,3'-di-O-glucoside	526133	262470	1.15	0.5	down
32	Mangiferonic acid	68634667	137513333	1.26	2	up
33	Trigonelline	22051667	45062000	1.27	2.04	up
34	Xanthosine	1051030	2140067	1.25	2.04	up
35	Orotic acid	743640	1551267	1.25	2.09	up
36	3-Hydroxypyridine	57370	121487	1.24	2.12	up
37	Fustin	3580267	7885433	1.25	2.2	up
38	4-Hydroxy-5-(2-oxo-1-pyrrolidinyl)benzoic acid	2657067	5872367	1.23	2.21	up
39	Coclaurine	46799000	104283333	1.23	2.23	up
40	10-Dehydrogeniposide	153079	341933	1.11	2.23	up
41	Quinic Acid	45742333	103103000	1.27	2.25	up
42	N-benzylformamide	3051533	6909533	1.25	2.26	up
43	N-Phenylacetylglycine	67666	154070	1.24	2.28	up
44	3-Oxooleana-11,13(18)-dien-28-oic acid	119751	275080	1.16	2.3	up
45	N-(2-Hydroxy-4-methoxyphenyl)acetamide	3694333	8568000	1.25	2.32	up
46	L-Tyrosine	8417033	19565667	1.25	2.32	up
47	N-Acetylisatin	757500	1760867	1.2	2.32	up
48	L-Ascorbic acid (Vitamin C)	85084667	211253333	1.26	2.48	up
49	Isocitric Acid	110618	274607	1.26	2.48	up
50	4-(3,4,5-Trihydroxybenzoxy)benzoic acid	20274667	51323667	1.27	2.53	up
51	D-Pantothenic Acid	4305933	11841000	1.27	2.75	up

52	Barbituric acid	4507700	12529000	1.26	2.78	up
53	Procyanidin A6 procyanidin	67774	204167	1.26	3.01	up
54	2-Phenylpropionic Acid	18693	61026	1.24	3.26	up
55	Tormentic Acid	44165	147107	1.21	3.33	up
56	LysoPC 16:0(2n isomer)	10427633	20999333	1.22	3.47	up
57	N-Acetyl-L-tyrosine	20387	294139	1.09	14.43	up

Table S7 unique differential metabolites in STZ vs MZ

NO	Metabolite	Chromatographic peak area		VIP	FC	type
		STZ	MZ			
1	N-Acetyl-L-Arginine	5320767	379173	1.06	0.07	down
2	N-Acetyl-L-Aspartic Acid	217477	40404	1.11	0.19	down
3	LysoPC 20:1	783760	171743	1.14	0.22	down
4	Quercetin-3-O-arabinoside (Guaijaverin)	571693	178443	1.26	0.31	down
5	Jujuboside B1	263590	91509	1.21	0.35	down
6	3-Cyano-L-alanine	295507	107214	1.22	0.36	down
7	L-Cyclopentylglycine	5169267	1842367	1.22	0.36	down
8	Vitexin-2''-O-rhamnoside	1205357	432653	1.17	0.36	down
9	3-Ureidopropionic Acid	15554667	5784700	1.21	0.37	down
10	O-Acetylserine	2040833	760827	1.25	0.37	down
11	Apiferol	665300	246570	1.18	0.37	down
12	Diosmetin-6-C-glucoside	167043	61667	1.18	0.37	down
13	2α,3α,19α-Trihydroxyursolic acid	257677	96014	1.17	0.37	down
14	Epigallocatechin	7637467	2906600	1.24	0.38	down
15	Phloretin	1124203	443883	1.22	0.39	down
16	LysoPC 19:2	177223	68260	1.2	0.39	down
17	Hispidulin-7-O-Glucoside	13580667	5468300	1.25	0.4	down
18	2'-Deoxyadenosine	1386200	573140	1.26	0.41	down
19	2α,3α,19α,23-Tetraydroxyurs-12-en-28-oic acid	212747	87540	1.06	0.41	down
20	LysoPE 20:2	166910	68111	1.09	0.41	down
21	Quercetin-3-O-rutinoside-7-O-rhamnoside	266603	109810	1.1	0.41	down
22	1-O-Galloyl-β-D-glucose	5553800	2327600	1.25	0.42	down
23	Kaempferol-3,7-O-dirhamnoside (Kaempferitrin)	53750667	22705667	1.21	0.42	down
24	3-Hydroxypropanoic acid	456870	195200	1.26	0.43	down
25	9-Hydroxy-12-oxo-10(E),15(	351163	150917	1.22	0.43	down

	Z)-octadecadienoic acid					
26	1-(sn-Glycero-3-phospho)-1D-myo-inositol	205856667	90421667	1.15	0.44	down
27	Diosmetin-7-O-glucoside	12223000	5380400	1.25	0.44	down
28	Epitheaflavic acid-3-O-Gallate	15120333	6778567	1.21	0.45	down
29	Procyanidin C1	5077900	2305800	1.15	0.45	down
30	1-(4-Methoxyphenyl)-1-propanol	1750567	812417	1.24	0.46	down
31	Chelidonic acid	28021667	13023333	1.21	0.46	down
32	4'-Hydroxy-5,7-dimethoxyflavanone	2455933	1122737	1.22	0.46	down
33	2,6-Diaminooimelic acid	507540	240760	1.23	0.47	down
34	Geniposide	395927	191597	1.1	0.48	down
35	Procyanidin C2	23926333	11453200	1.18	0.48	down
36	N6-Acetyl-L-lysine	1837733	901160	1.16	0.49	down
37	Orientin-7-O-glucoside	8074033	3970967	1.25	0.49	down
38	Hydroxytyrosol	1641000	813707	1.26	0.5	down
39	LysoPC 12:0	17067333	34697667	1.21	2.03	up
40	Dihydroferuloylputrescine	182820	373397	1.23	2.04	up
41	6-Methoxykaempferol-3-O-glucoside	1937100	3980633	1.12	2.05	up
42	Tamarixetin-3-O-rutinoside	776470	1593933	1.18	2.05	up
43	N-Glycyl-L-leucine	189600	391673	1.24	2.07	up
44	D-Threonic Acid	6786067	14316333	1.1	2.11	up
45	Esculetin	234837	510800	1.2	2.18	up
46	O-Nornuciferine (N-Methylasimilobine)(Floribundine)	272847	595047	1.23	2.18	up
47	9,12,13-Trihydroxy-10,15-octadecadienoic acid	552817	1243633	1.24	2.25	up
48	1-O-Caffeoyl-β-D-glucose	1891667	4283600	1.25	2.26	up
49	1-Linoleoylglycerol	57142	129330	1.2	2.26	up
50	Crepenynic acid	643990	1468033	1.22	2.28	up
51	Isorhamnetin-3-O-rutinosid	1992567	4564633	1.21	2.29	up

	e (Narcissin)					
52	γ-Linolenic Acid	612657	1427133	1.24	2.33	up
53	Isorhamnetin-3-O-Glucoside	186543	454333	1.18	2.44	up
54	Cis-4,7,10,13,16,19-Docosahexaenoic Acid	249560	615857	1.23	2.47	up
55	N-Acetyl-L-phenylalanine	890820	2417500	1.25	2.71	up
56	Quercetin-3-O-sophoroside (Baimaside)	577727	1647233	1.25	2.85	up
57	Quercetin-3-O-(6''-O-acetyl) galactoside	194133	750793	1.21	3.87	up
