# Supplementary figures and images for: Analysis of Metabolites and Metabolic Pathways of Three Chinese Jujube Cultivar
Source: Metabolites. 2023 May 31;13(6):714. doi: 10.3390/metabo13060714 (PMC10305511; doi:10.3390/metabo13060714)

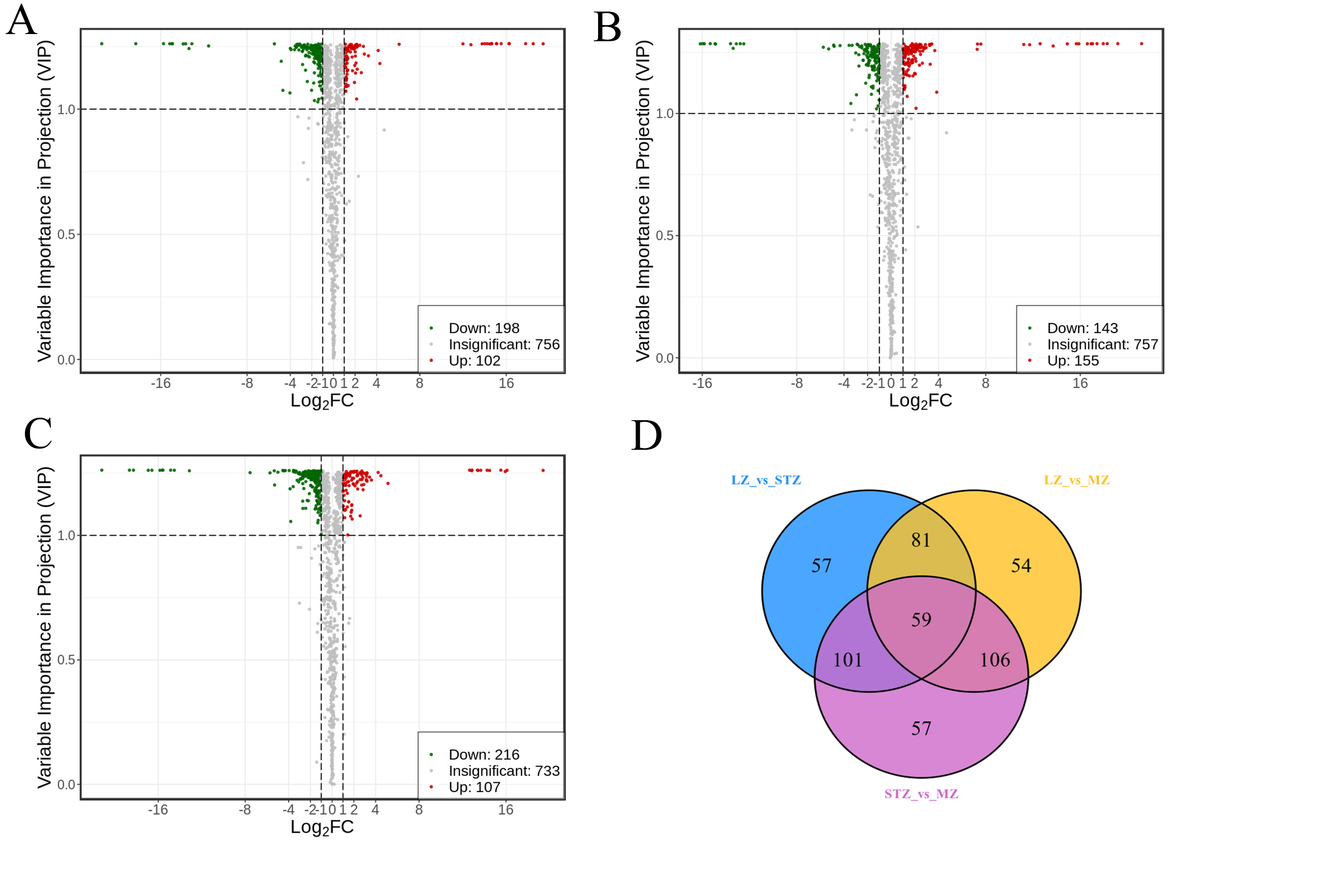

Supplement: Supplementary file 1 [file metabolites-13-00714-s001.zip › Fig S1.jpg]
